# MiR-29a Increase in Aging May Function as a Compensatory Mechanism Against Cardiac Fibrosis Through SERPINH1 Downregulation

**DOI:** 10.3389/fcvm.2021.810241

**Published:** 2022-01-18

**Authors:** Evelyn Gabriela Rusu-Nastase, Ana-Mihaela Lupan, Catalina Iolanda Marinescu, Carmen Alexandra Neculachi, Mihai Bogdan Preda, Alexandrina Burlacu

**Affiliations:** Laboratory of Stem Cell Biology, Institute of Cellular Biology and Pathology “Nicolae Simionescu”, Bucharest, Romania

**Keywords:** aging, miRNA, fibrosis, SERPINH1, prediction analysis, miR-29a

## Abstract

Deregulation of microRNA (miRNA) profile has been reportedly linked to the aging process, which is a dominant risk factor for many pathologies. Among the miRNAs with documented roles in aging-related cardiac diseases, miR-18a, -21a, -22, and -29a were mainly associated with hypertrophy and/or fibrosis; however, their relationship to aging was not fully addressed before. The purpose of this paper was to evaluate the variations in the expression levels of these miRNAs in the aging process. To this aim, multiple organs were harvested from young (2–3-months-old), old (16–18-months-old), and very old (24–25-months-old) mice, and the abundance of the miRNAs was evaluated by quantitative real-time (RT)-PCR. Our studies demonstrated that miR-21a, miR-22, and miR-29a were upregulated in the aged heart. Among them, miR-29a was highly expressed in many other organs, i.e., the brain, the skeletal muscle, the pancreas, and the kidney, and its expression was further upregulated during the natural aging process. Western blot, immunofluorescence, and xCELLigence analyses concurrently indicated that overexpression of miR-29a in the muscle cells decreased the collagen levels as well as cell migration and proliferation. Computational prediction analysis and overexpression studies identified SERPINH1, a specific chaperone of procollagens, as a potential miR-29a target. Corroborating to this, significantly downregulated SERPINH1 levels were found in the skeletal muscle, the heart, the brain, the kidney, and the pancreas harvested from very old animals, thereby indicating the role of the miR-29a-SERPINH1 axis in the aging process. *In vitro* analysis of miR-29a effects on fibroblast and cardiac muscle cells pointed toward a protective role of miR-29a on aging-related fibrosis, by reducing cell migration and proliferation. In conclusion, our study indicates an adaptive increase of miR-29 in the natural aging process and suggests its role as a transcriptional repressor of SERPINH1, with a potential therapeutic value against adverse matrix remodeling and aging-associated tissue fibrosis.

## Introduction

The persistent increase in human longevity has stimulated the interest of scientists in understanding the fundamental mechanisms underlying natural aging, which is the dominant risk factor for many pathologies, including cardiovascular diseases (1). Aging is a progressive process characterized by gradual changes and alterations accumulated in the tissues and organs, which eventually results in the reduced ability of the organism to meet increased demands (2, 3). The alterations associated with the aging process can be grouped into several hallmarks of aging, such as genomic instability, telomere attrition, epigenetic alterations, loss of protein homeostasis, deregulated nutrient sensing, mitochondrial dysfunction, cellular senescence, stem cell exhaustion, and altered intercellular communication (4). Thus, during aging, the accumulation of somatic mutations in the nuclear DNA may affect the essential genes and transcriptional pathways that may compromise tissue homeostasis (4). Besides, natural aging is associated with telomere attrition, which may promote the development of degenerative defects, such as pulmonary fibrosis or dyskeratosis congenita (5, 6). Aging is also associated with increased production of reactive oxygen species, which are known to induce damages in mitochondrial DNA and redox-sensitive mitochondrial proteins, and to promote mitochondrial dysfunction, thus leading to heart failure and other pathologies (7). All these hallmarks are interwoven and promote the gradual loss of physiological integrity and cell function, and eventually lead to the death of the organism.

During aging, the cardiac tissue architecture deteriorates, leading to a gradual decline in the cardiac reserves, a major pathophysiological feature of the heart in old individuals (8). Cardiac fibrosis and hypertrophy are two important hallmarks of an aged heart and they are tightly interwoven and mutually triggered by each other. Thus, the baseline activation of fibrotic pathways in the aged heart is followed by defective scar formation and left ventricular hypertrophy, making the heart prone to adverse dilative remodeling. Consequently, cardiac aging is associated with diastolic dysfunction and heart failure with preserved systolic function, which has significant impacts on the elderly population. Dissecting the mechanisms of interstitial fibrosis and cardiomyocyte hypertrophy in the aging heart is therefore critical in order to inspire new approaches for the prevention of adverse remodeling and heart failure in old patients.

MicroRNAs (miRNAs) are a class of small non-coding RNAs that regulate gene expression at the posttranscriptional level and together with other non-coding RNA molecules, are powerful regulators of a wide range of biological mechanisms in many disorders (9, 10). Notably, numerous basic and clinical studies have connected miRNA to the initiation and/or progression of hypertrophy and fibrosis (11, 12), which make them exciting potential therapeutic targets in these conditions.

One of the most influencing miRNAs in the cardiovascular system is miR-21, which is the major miRNA in cardiac fibroblasts, where it accounts for around 20% of total miRNAs (13). MiR-21 was found increased in the naturally aged murine hearts (14), as well as in plasma samples from centenarians (15). Besides, various animal models of cardiac fibrosis (16), hypertrophic heart (17), and heart failure (18) identified the presence of miR-21 in increased amounts in comparison to control groups, being related to cardiac dysfunction. This miRNA has attracted attention for its diverse effects in cardiac function (19) and was extensively reported to exert both protective and harmful effects, being involved in different cell processes, such as cell growth and proliferation, mitochondrial damage, chemosensitivity, response to stress, and cell invasion (15). In accordance to this, the dysregulation of miR-21 occurred in diverse pathologies, like cancer and metabolic disorders (20, 21).

MiR-17-92 cluster, which consists of six mature miRNAs (miR-17, miR-18a, miR-19a, miR-19b, miR-20a, and miR-92a), has also been involved in cardiac aging. Within this cluster, miR-18a was reported to regulate the expression of several extracellular matrix proteins (22) and its decreased expression in heart failure in the aged individuals was linked to aging-induced heart remodeling. Corroborating to this, downregulation of miR-18a was associated with increased fibrosis and a decline in heart function (23), while over-expression of miR-18a reduced fibrosis, hypertrophy, and the apoptosis of cardiomyocytes in heart failure (24).

Another miRNA involved in cardiac aging is miR-22. Previous studies reported a prohypertrophic effect of miR-22, with a critical role in cardiac remodeling (25–27). This miRNA was found increased in aging hearts and this upregulation was linked to accelerated senescence and increased migration of cardiac fibroblasts (28). Corroborating to this, the inhibition of miR-22 stimulated cardiac autophagy and enhanced cardiac function in older mice with myocardial infarction (29).

Another miRNA with a prominent role in cardiac hypertrophy and fibrosis is miR-29a, which is the most stable and most abundantly expressed member of its family (30). MiR-29 is an essential regulator of extracellular matrix proteins in pathways related to fibrosis (31, 32) and has been reported to be dramatically downregulated in the region of the fibrotic scar after myocardial infarction (33). Recent studies have shown that miR-29a has different roles in cardiac fibroblasts, where it inhibits collagen synthesis, thus attenuating fibrosis (33) and cardiomyocytes, which induces hypertrophy. Overall, the deregulation of miR-29a was associated with conditions, such as myocardial fibrosis, cardiac hypertrophy, congestive heart failure, chronic hepatic injury, hepatitis C virus (HCV) infection and inflammation, hypertensive and diabetic nephropathies, and chronic kidney diseases (30).

Aiming at identifying miRNAs with therapeutic value for the cardiac fibrosis associated with the natural aging process, we explored whether the expression of these four miRNAs changed during the natural aging process. Here, we report that miR-29a increase is a common feature of multiple organs in the natural aging process and it may represent a compensatory mechanism against cardiac fibrosis through SERPINH1 downregulation.

## Materials and Methods

### Animals

All animal experiments were conducted in accordance with the European Guidelines for Animal Welfare (Directive 2010/63/EU) and approved by the National Sanitary Veterinary and Food Safety Authority (nr 389/22.03.2018). C57Bl/6 mice were purchased from Jackson Laboratory and bred in the ICBP animal facility, under specific pathogen-free conditions, in a controlled environment of 12/12 h light/dark cycle, 21°C, 55–60% humidity, with chow and water ad libitum. Female and male C57BL/6J mouse cohorts used in this study were divided into three age groups: young (2–3 months-old, *n* = 20), old (16–18 months-old, *n* = 4), and very old (24–25 months-old, *n* = 10).

### Cell Culture

The NIH/3T3 and HL-1 cell lines were purchased from ATCC and Merck Millipore, MA, USA, respectively. NIH/3T3 cells were cultured in high-glucose DMEM (Thermo Fisher Scientific, MA, USA), supplemented with 10% fetal bovine serum (FBS) (Thermo Fisher Scientific, MA, USA) and 1% antibiotic-antimycotic solution (Merck KGaA, Darmstadt, Germany) and passaged two times a week at a density of 3 × 10^3^ cells/cm^2^. HL-1 cells were cultured in Claycomb medium supplemented with 10% FBS, 1% non-essential amino acids (NEAA), 0.1 mM norepinephrine, and 2mM L-glutamine and passaged every three days by 1:3 splitting onto gelatin/fibronectin-coated tissue flasks.

Primary cardiac fibroblasts were isolated from mouse ventricles as previously described (13). Briefly, the mice were anesthetized with ketamine: xylazine (100:10, mg/kg b.w.); then, the hearts were removed, rinsed with ice-cold phosphate-buffered saline (PBS), and minced into 1- to 2-mm pieces under a sterile laminar flow hood. The fresh tissue was dissociated into a single-cell suspension through 5-min-sequential steps using 5 ml Hanks' balanced salt solution (HBSS) with 0.1% trypsin and 100 μg/ml type I-S collagenase (Merck KGaA, Darmstadt, Germany). Around 5–8 fractions were collected until the whole tissue was digested. Each collected fraction was mixed with 2 ml FBS before centrifugation at 400 × *g*, 4°C for 5 min. In the end, all cellular pellets were pooled and resuspended in 10 ml of Dulbecco's Modified Eagle's Medium (DMEM)/F12 supplemented with 10% FBS, 1% antibiotic-antimycotic solution, 1 × L-glutamine (Merck KGaA, Darmstadt, Germany), 10 ng/μl fibroblast growth factor (FGF) (R&D Systems, Canada), and 10 μg/ml insulin for plating. Cells were allowed to adhere and proliferate in culture for 5 days before being used in experiments.

### Tissue Sampling

Mice were anesthetized with ketamine: xylazine (100/10 mg/kg b.w.). The organs were harvested as follows: the cardiac ventricle, the mediastinal lymph nodes, the thymus, a portion of the right liver lobe, the spleen, the pancreas, the left kidney, the left quadriceps muscle, and the whole brain. The lymphatic organs were harvested only from young and old mice. The organs were immediately flash-frozen in liquid nitrogen in 2-ml RNase-free tubes and stored at −80°C. Organ powder was obtained from each collected organ by grinding in liquid nitrogen using a mortar and pestle and stored at −80°C until use.

### Total RNA Preparation

The total RNA was extracted from 10 mg frozen organ powder in 1 ml of Trizol® reagent (Thermo Fisher Scientific, MA, USA) with phenol/chloroform, by following the manufacturer's recommendations. For RNA isolation from cardiac fibroblasts, the miRNeasy kit (Qiagen, MD, USA), as per the protocol recommended by the manufacturer, was used. Total RNA was eluted in 30 μl of nuclease-free H_2_O and stored at −80°C until use.

### Quantification of miRNAs Using RT-qPCR

For quantification of mature miRNA strands, 25 ng of the total RNA were reverse-transcribed to complementary DNA (cDNA) using TaqMan® MiRNA assays and Applied Biosystems® TaqMan® MiRNA Reverse Transcription Kit. The reverse transcription was carried out on a Veriti™ 96-Well Thermal Cycler and the real-time quantitative reverse transcription PCR (RT-qPCR) was carried out using 0.2 μl primers and TaqMan probe, at a final reaction volume of 10 μl, on ViiA™ 7 RT-PCR System. The cycling conditions were as follows: enzyme activation at 95°C for 20 s, denaturation at 95°C for 1 s, and annealing/extension at 60°C for 20 s. Forty cycles of amplification were performed.

### Real-Time RT-qPCR Analysis

The reverse-transcription reaction was made with 500 ng total RNA, using the High-Capacity cDNA Reverse Transcription Kit (Thermo Fisher Scientific, MA, USA). Quantification of SERPINH1 was carried out using SYBR™ Select Master Mix (Thermo Fisher Scientific, MA, USA) and ViiA™ 7 Real-Time PCR System. Relative expression was calculated using the comparative C_T_ method and mouse S18 was used for normalization.

### Cell Transfection With miR-29a Mimic

Cells were seeded onto a 24-well plate at 70% confluence, one day prior to transfection. Cells were transiently transfected with mature miR-29a-3p mimic (Thermo Fisher Scientific, MA, USA, ID MC12499) or scrambled miRNA control (negative control) (Thermo Fisher Scientific, MA, USA, Cat. 4464058) at a final concentration of 10 nM, using Lipofectamine™ RNAiMAX Transfection Reagent (Thermo Fisher Scientific, MA, USA). The medium was replaced with a complete growth medium after 6 h of transfection, and cells were maintained in culture for another 24 h, before cellular assays.

### Target Prediction and Functional Analysis of miR-29a

The miR-29a targets were predicted using three online analysis tools, such as TargetScan (34), DIANA-microT-CDS (35), and miRDB (36). To reduce possible false positives, only the overlapped predicted targets of at least two databases were considered for functional analysis. Biological process enrichment analysis was performed using ClueGO v2.5.7 (37), a plug-in of Cytoscape v3.8.2 (38), and the criteria for grouping the genes for each term were as follows: 24 genes per term, 10% genes per term with a kappa score threshold of 0.4, minimum GO level of 3, and maximum GO level of 8. The enrichment/depletion test for terms was set to two-sided (enrichment/depletion) based on a hypergeometric test, and the corrected method for *p*-value was set as per the Benjamini-Hochberg method. The adjusted *p* < 0.01 was considered statistically significant.

### Protein-Protein Interaction (PPI) Analysis

For PPI analysis, the target genes were mapped to the STRING v11 database (39) and only the interactions with a combined score higher than 0.4 and FDR stringency less than 1% were considered significant. The network file was loaded in Cytoscape software for visual adjustment. To emphasize the relationships between proteins, the size of nodes was correlated with the number of interactions of each particular protein, and the thickness of edges was correlated with the combined score.

### Hydroxyproline Assay

Quantification of total collagen level in the cardiac ventricle was performed using a Hydroxyproline Assay Kit (Merck KGaA, Darmstadt, Germany, MAK008) and following the manufacturer's recommendations. Briefly, 10 mg wet tissue was homogenized in 100 μl of water and hydrolyzed with 100 μl concentrated HCl for 3 h at 120 °C. The mixture was centrifuged and 30 μl supernatant was transferred into a 96-well plate and evaporated to dryness in a 60°C oven. The oxidation reaction was done using Chloramine T for 5 min at room temperature, and then the samples were mixed with DMAB/perchloric acid/isopropanol reagent and incubated for 90 min at 60°C. Hydroxyproline was measured spectrophotometrically at 560 nm using a standard curve.

### Immunofluorescence Assay

Cells were fixed in 4% formaldehyde, permeabilized with 0.1 % TritonX100, and incubated with anti-Collagen I (Novus Biologicals, CO, USA, NB600-408-1, dilution 1: 50) or anti-Collagen III (Abcam, Cambridge, UK, ab7778, dilution 1: 100) rabbit polyclonal antibody, overnight at 4 °C. Washed cells were then incubated with anti-rabbit IgG (whole molecule)-FITC secondary antibody (Merck KGaA, Darmstadt, Germany, F9887, dilution 1: 320) for 1 h at room temperature in the dark. Image acquisition was performed using a Leica DMi8 inverted fluorescent microscope (Leica Microsystems GmbH, Wetzlar, Germany) equipped with HC PL APO 20x/0.75 CS2 dry objective. Fluorophores were excited with a multi-LED Spectra-X light source (Lumencor, OR, USA) and images were captured with an sCMOS camera Leica DFC9000 and subsequently processed with Leica LAS X software. Z-stack images (15-μm depth) were acquired with LAS X Navigator module and mosaic merged and then digitally processed for extended depth of field. The fluorescence was quantified with the analysis tool of Las X software by averaging the least four random fields per each experimental condition and the results were expressed as mean fluorescence intensity.

### Western Blot Analysis

Cells were lysed in 2X Laemmli buffer containing proteases inhibitor cocktail (Merck KGaA, Darmstadt, Germany), and protein concentration was determined using the Amido Black assay. A total of 40 μg of protein was loaded and resolved on 4–15% of precast polyacrylamide gel, followed by a transfer on 0.45 μm nitrocellulose membrane and Ponceau S reversible staining. After blocking 3 h in 5% skim milk in Tris-buffered saline containing 0.1% Tween 20 (TBS-T), the membranes were incubated with anti-collagen III antibody (Abcam, Cambridge, UK, ab7778, 1 μg/ml) overnight at 4°C. Membranes were rinsed in TBS and further incubated with HRP-conjugated secondary antibody (R&D Systems, Canada, HAF008) for 1 h. Protein bands were imaged after enhancement with a chemiluminescence agent (Thermo Fisher Scientific, MA, USA) using an ImageQuant LAS4000 system (Fujifilm, Minato, Japan). The protein expression was quantified by densitometry with TotalLab software and normalized to total protein as quantified by Ponceau S staining.

### Analysis of Cell Migration and Proliferation Using xCELLigence System

The effect of miR-29a overexpression on HL-1 cell proliferation was evaluated with the xCELLigence System (Agilent Technologies, CA, USA). This system monitors cellular events in real-time by using the microelectrodes integrated at the bottom of the specially designed E-plates. Briefly, 24 h after transfection initiation, the cells (expressing scramble miRNA sequence or miR-29a mimic) were seeded onto E-plates (15 × 10^3^ cells/well) in a complete medium, and the cell index was recorded at 1-h interval for up to 48 h. The migratory capacity of the transfected cells was evaluated using CIM-plates, which are double-chamber systems (with 8-μm pore membranes) bearing microelectrodes onto the inner face of the membrane. Briefly, the cell suspension prepared in serum-free medium was added to the upper chamber (40 × 10^3^ cells/well) and the cells were allowed to migrate through the membrane toward the lower chamber, containing the complete medium. When cells pass through the membrane in response to serum, they come in contact with the microelectrodes and generate impedance signals.

### Cell Cycle Analysis

Cell proliferation was analyzed using Propidium iodide staining and flow cytometry analysis. Briefly, the cell suspension containing 10^5^ cells was fixed with 70% ice-cold ethanol (3 h at −20°C) and then incubated with 20 μg/ml propidium iodide and 100 μg/ml RNAse A. The stained cells were analyzed with a CytoFlex flow cytometer (Beckman Coulter, CA, USA). At least 20,000 events were analyzed per sample. The different phases of the cell cycle (G1, S, and G2/M) were determined based on the fluorescent peak signal, which was proportional to cellular DNA content.

### Statistical Analysis

All results were expressed as mean +/– SD. Statistical analysis was performed using GraphPad Prism 9.1.2 (GraphPad Software Inc, CA, USA). *P*-value has been calculated using unpaired *t*-test for equal variances and unpaired *t*-test with Welch's correction for unequal variances. For cell cycle experiments, two-way ANOVA was used. Correlations were done using Pearson's correlation coefficient, for normally distributed data, or Spearman's correlation coefficient, for non-normally distributed data. A *p*-value less than 0.05 was considered statistically significant.

## Results

### Age-Related Changes in the Expression Levels of miR-18a, miR-21a, miR-22, and miR-29a in the Heart and the Skeletal Muscle of C57Bl/6J Mice

**Figure 1:**
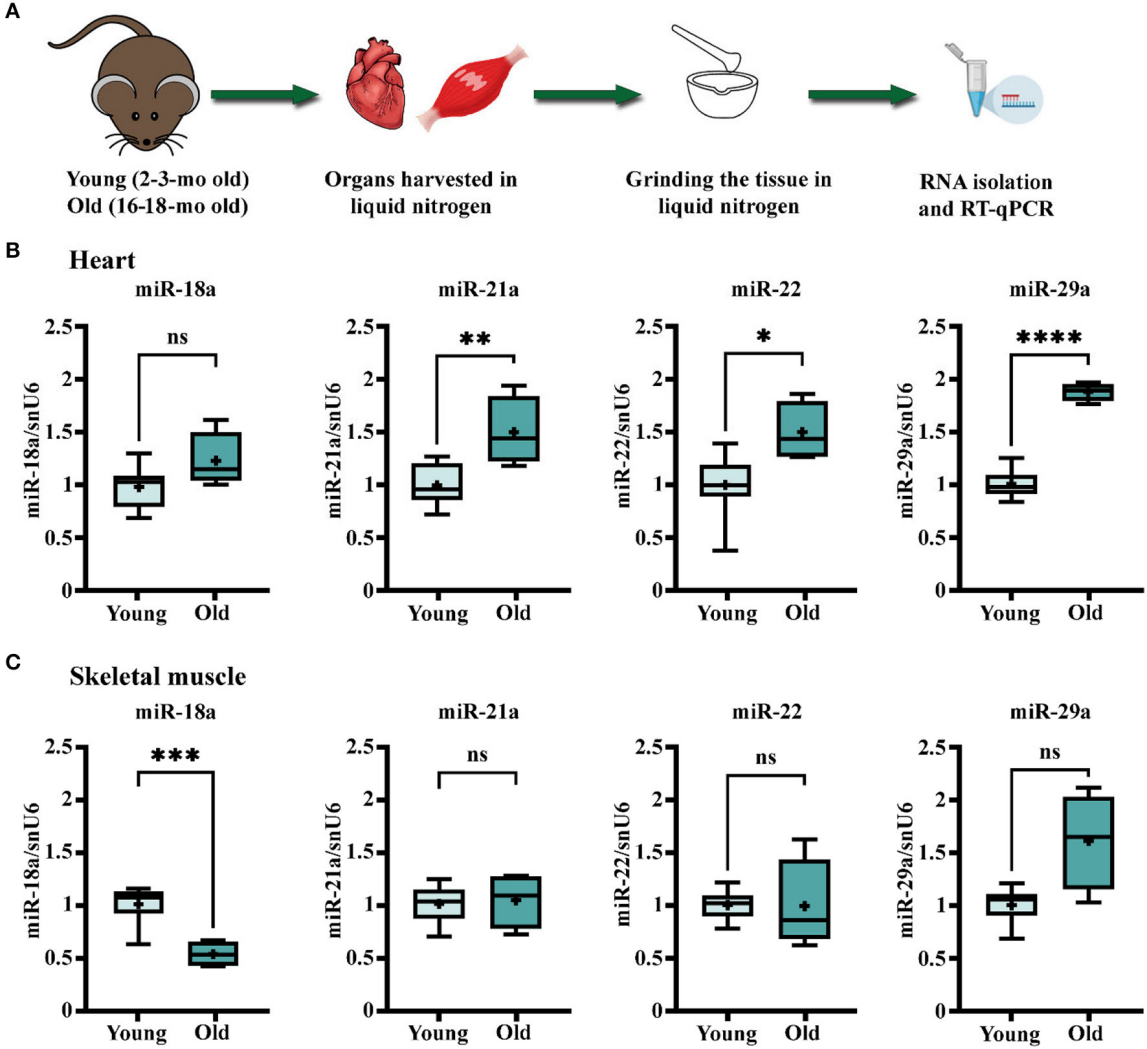
Age-related changes in the expression levels of miR-18a, miR-21a, miR-22, and miR-29a in heart and skeletal muscle of C57Bl/6J mice. **(A)** Schematic illustration of the experimental design. The heart ventricles and left quadriceps muscle were harvested from young (*n* = 10) and old (*n* = 4) mice and processed for total RNA isolation and real-time quantitative reverse transcription PCR (RT-qPCR) analysis. **(B)** The gene expression level of the four miRNAs in the heart. **(C)** The gene expression level of the four miRNAs in the skeletal muscle. *p < 0.05, **p < 0.01, ***p < 0.005, and **** *p* < 0.001.

To address whether the aging process impacts the abundance of these miRNAs in the myocardium, the expression levels were evaluated in the heart ventricle and the skeletal muscle of young (2–3-months-old) and old (16–18-months-old) animals (Figure 1A). The rationale of introducing skeletal muscle samples in the analysis was to help discern the cardiac specificity of miRNA changes. The results showed that miR-21a, -22, and -29a had significantly higher levels in old mice-derived cardiac muscle (Figure 1B). Among them, miR-29a had a tendency of increased expression in the skeletal muscle of old animals as well (Figure 1C), while miR-21a and miR-22 were not modified (Figure 1C), thus suggesting the probability of heart-specific changes for miR-21a and miR-22 during the natural aging process. On the contrary, miR-18a had different age-associated patterns, with constant expression in the old ventricle and decreased expression in the old skeletal muscle.

### Age-Related Changes in the Expression Levels of miR-21, miR-22, and miR-29a in Mouse Lymphoid Organs

**Figure 2:**
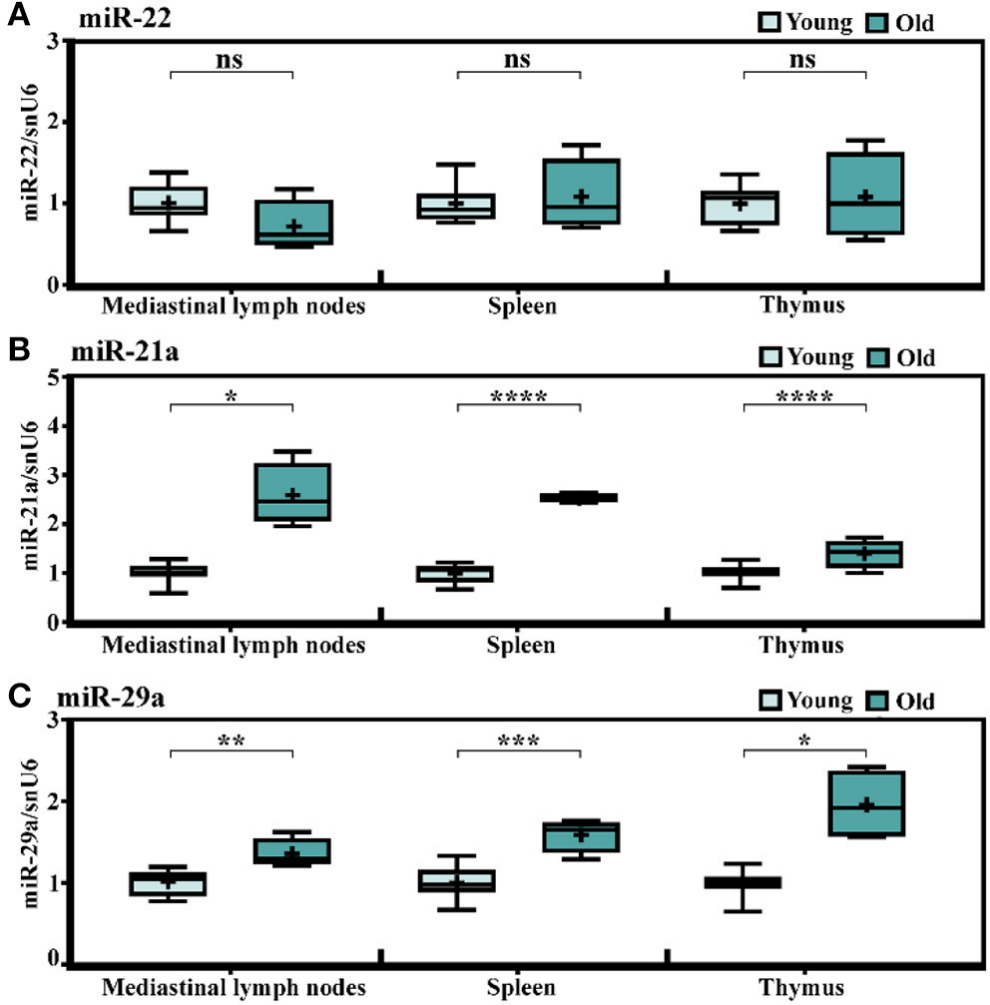
Age-related changes in the expression levels of miR-21a, miR-22, and miR-29a in lymph organs of C57Bl/6 mice. The expressions of **(A)** miR-22, **(B)** miR-21a, and **(C)** miR-29a in the mediastinal lymph nodes, the spleen, and the thymus harvested from the young (*n* = 10) and old (*n* = 4) animals. The results are presented as fold changes relative to the young group. *p < 0.05, **p < 0.01, ***p < 0.005, ****p < 0.001.

In an attempt to find out whether the immune cells infiltrating the old heart might be the source of the increased miRNA levels, the expression levels of miR-21a, -22, and -29a were determined in the mediastinal lymph nodes (as the heart draining lymph nodes), the spleen, and the thymus, knowing that the chronic low-grade systemic inflammation is a key pathological characteristic of aged myocardium. The qRT-PCR analysis showed no age-dependent alteration of miR-22 levels in the lymphoid organs in old animals (Figure 2A), and generally, there were very low expression levels of this miRNA in the three organs (data not shown). These results excluded the contribution of the lymphoid organs to the miR-22 increase in the old heart and further suggested that the age-associated miR-22 increase might be confined to the heart. Indeed, gene expression analysis of miR-22 in other organs harvested from different experimental lots including young (2–3-months-old) and very old (24–25-months-old) animals confirmed the steady expression levels in the kidney, the liver, and the brain (Supplementary Figure 1). It is however uncertain which cell type might be the source of miR-22 increase in the cardiac tissue as neither miRNA sequencing (GEO accession number GSE153214) nor qRT-PCR analysis identified any change in the expression level of this miRNA in isolated populations of cardiac fibroblasts and cardiomyocytes (data not shown). Interestingly, both miR-21a and miR-29a had significantly higher expressions in the heart draining lymph nodes in old animals, as well as in the spleen and the thymus (Figures 2B,C), which make them more conceivable in the hypothesis of the infiltrating immune cells into the aged heart as contributors to the increase in these miRNA levels and also advances these modifications as being common features of aging tissues.

### The Age-Related Variations of miR-21a and miR-29a Levels in Various Organs

**Figure 3:**
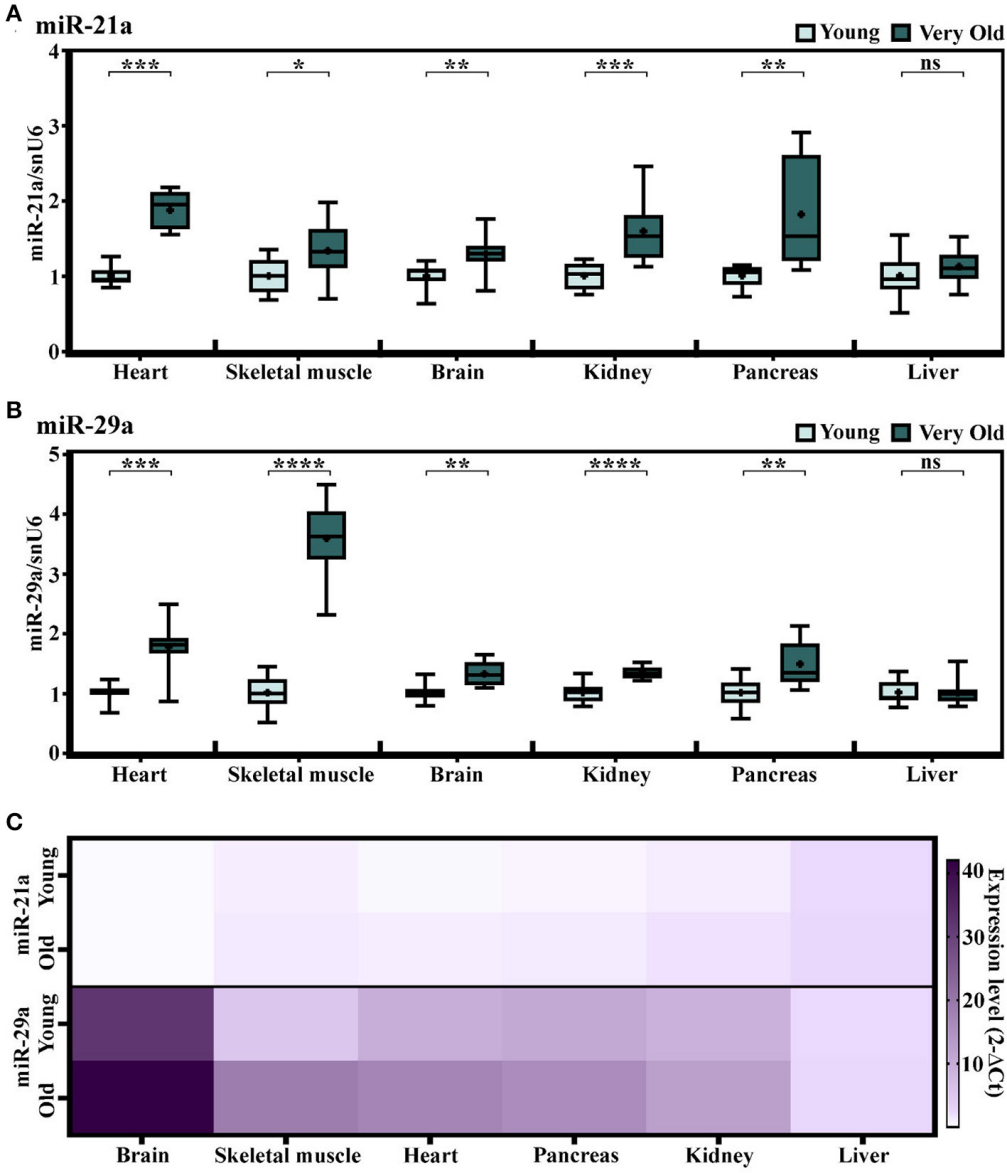
**(A,B)** The expression levels of miR-21a **(A)** and miR-29a **(B)** in multiple organs harvested from young (2–3-months-old) and very old (24–25-months-old) mice. The results are presented as fold changes relative to the young group per each organ (the heart, the skeletal muscle, the brain, the kidney, the pancreas, and the liver); *n* = 10 animals/group. *p < 0.05, **p < 0.01, ***p < 0.005, and ****p < 0.001. **(C)** Heat map showing the expression of miR-21 and miR-29a normalized to snoRNA/U6 in each organ. Note the higher expression of miR-29a in comparison to miR-21 in the analyzed organs.

To further investigate whether the increases in miR-21a and miR-29a expression levels in the natural aging process are general aging features, several other organs have been included in the analysis. Having the steady or not-significantly increased expressions of miR-21a and miR-29a in the skeletal muscle of old animals (16–18-months-old) in comparison to young counterparts (described in Figure 1C), we attempted to investigate the miRNA expressions at even older ages, by establishing another experimental lot comprising young (2–3-months-old) and very old (24–25-months-old) animals. Multiple organs have been included in the analysis i.e., the cardiac ventricle, the skeletal muscle, the brain, the kidney, the pancreas, and the liver. The results confirmed the aging-induced increase of miR-21a expression in the cardiac ventricle and also showed significant increases in all the other analyzed organs, except the liver (Figure 3A). However, apart from the heart, the increases in the other organs were minor and probably without biological significance.

Similarly, miR-29a showed statistically significant increases in the heart, the skeletal muscle, the brain, the kidney, and the pancreas, yet not in the liver of the old animals (Figure 3B). Nevertheless, in contrast to miR-21a, the increase in miR-29a was higher in the skeletal muscle and its expression level had a gradually increasing tendency during aging, which was more obvious than in the cardiac ventricle (Supplementary Figure 2). Supporting this, the miRNA sequencing of cardiac fibroblasts isolated from the young and old mice (13) showed slightly increased levels of miR-29a (Supplementary Figure 3), which could not be further validated by qRT-PCR analysis. It is also worth mentioning that, although highly abundant in the cardiac fibroblasts, miR-21a was not found altered in old cardiac fibroblasts (Supplementary Figure 3) and, furthermore, its levels were lower than those of miR-29a in either the organ investigated from the young or the old individuals (Figure 3C). Together, these data suggest that the aging-related increase in miR-29a, although apparently a feature of multiple organs, is more obvious in muscle tissues, with putative contributors being the infiltrating immune cells, rather than the resident cardiac cell populations.

### Validation of SERPINH1 as a Direct miR-29a Target

**Figure 4:**
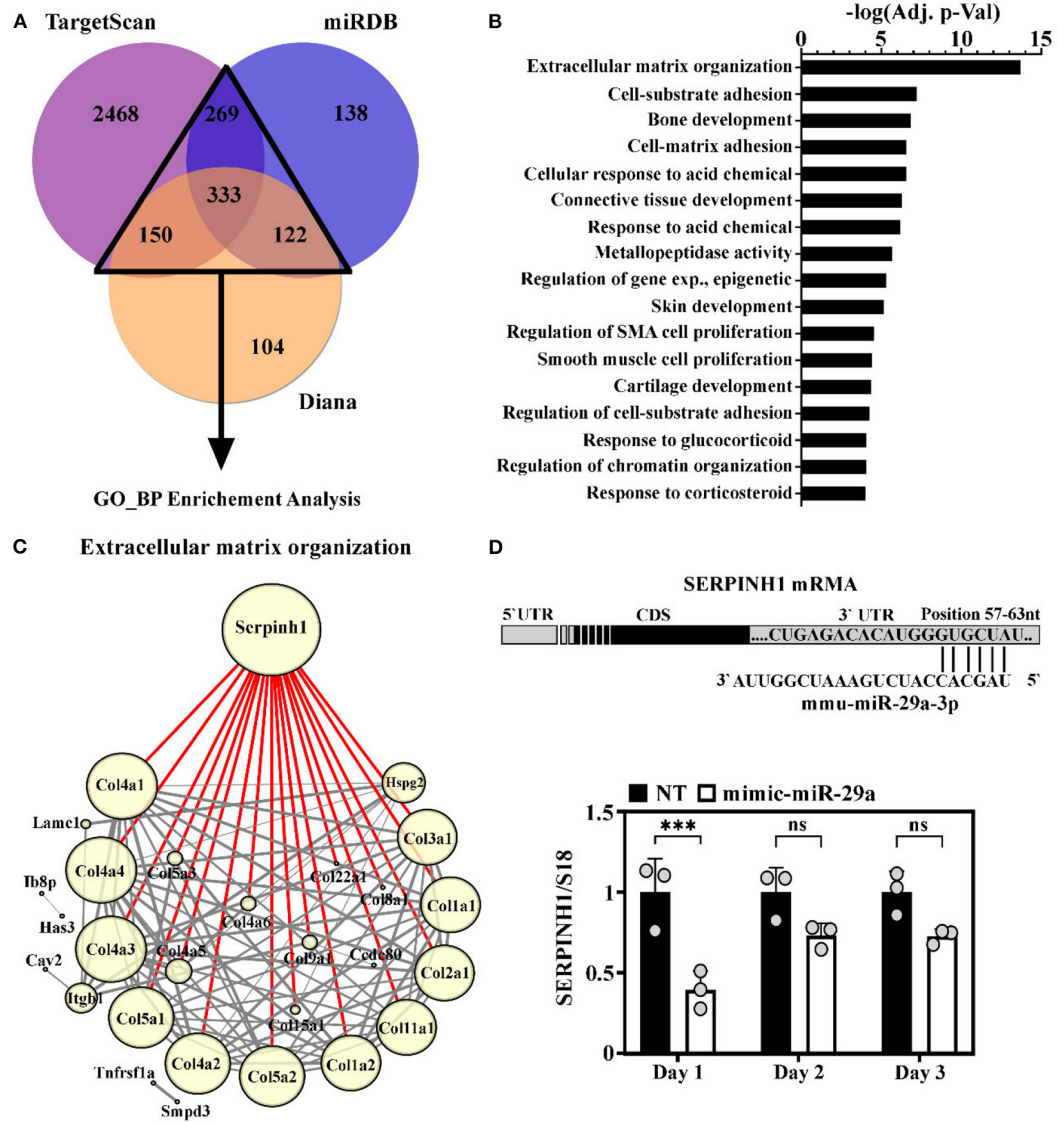
Identification of SERPINH1 as a miR-29a target. **(A)** Venn diagram showing the predicted targets of the miR-29a, obtained by interrogating three computational algorithms i.e., TargetScan, miRDB, and Diana. The targets predicted by at least 2 databases (with numbers illustrated in a triangle) were used in gene ontology (GO) analysis. **(B)** Enrichment analysis of predicted miR-29a target genes. Note that extracellular matrix (ECM) organization emerged as the most significantly enriched process involving miR-29a target genes. **(C)** String analysis of genes involved in ECM organization. Node size directly correlates with the number of interactions of each protein (min = 1; max = 18); Edge width denotes the confidence score calculated by STRING: the thinnest = 0.4 (medium confidence); the thickest ≥ 0.9 (the highest confidence); Highlighted in red are the interaction of SERPINH1. **(D)** Illustration of the 3′ UTR region of the mouse SERPINH1 gene with the predicted site for miR-29a (below). The expression of SERPINH1 mRNA level in HL-1 cells at one, two, and three days after transient transfection of cells with miR-29a mimic; ***p < 0.005.

The potential impact of miR-29a increase during the aging process was investigated through bioinformatic analysis of the targets predicted by three databases. Concisely, the targets predicted by at least two of the three databases (TargetScan v7.2, miRDB, and DIANA v5.0) were used for Gene Ontology (GO)-Biological Processes enrichment analysis (Figure 4A). This analysis highlighted extracellular matrix (ECM) organization as the most significantly enriched GO term. This result was in accordance with the ability of miR-29 to regulate a broad collection of mRNAs with roles in fibrosis and ECM remodeling (33). Other enriched GO terms mostly included biological processes involved in connective tissue homeostasis, e.g., cell-matrix adhesion, metallopeptidase activity, regulation of smooth muscle cell proliferation, etc (Figure 4B).

To better understand the relationship between the predicted targets implicated in ECM organization, the PPI analysis was done using the STRING database. To visualize this network, the 46 genes included in this term were imported into Cytoscape. The generated network was further processed by eliminating the proteins with no direct interactions among them. The final network, containing 28 nodes and 188 interactions, is illustrated in Figure 4C. The node size correlates with the number of interactions between proteins and the edge thickness correlates with the confidence score. This analysis highlighted Serpinh1 as the most important component in the network. SERPINH1 interacts with high confidence scores (> 0.9) with 18 proteins, all from the collagen family (Figure 4C).

To experimentally validate miR-29a as a modulator for SERPINH1 in the muscle cells, the gain-of-function approach was used in HL-1 cardiac muscle cells by transient transfection with miR-29a mimic, which resulted in a marked increase of the endogenous expression level of miR-29a, peaking at 1 day after oligonucleotide addition (Supplementary Figure 3). The RT-qPCR analysis of SERPINH1 in the transfected cells showed that the overexpression of miR-29a caused a pronounced reduction of SERPINH1 (2.5-fold decrease) at the mRNA level at 1 day after transfection (Figure 4D). Similar results were obtained with the 3T3 fibroblasts cell line (Supplementary Figure 5). This data validates SERPINH1 as a direct target of miR-29a in cardiac muscle cells.

### Increased miR-29a Expression Inversely Correlates With SERPINH1 in Multiple Organs in Old Mice

**Figure 5:**
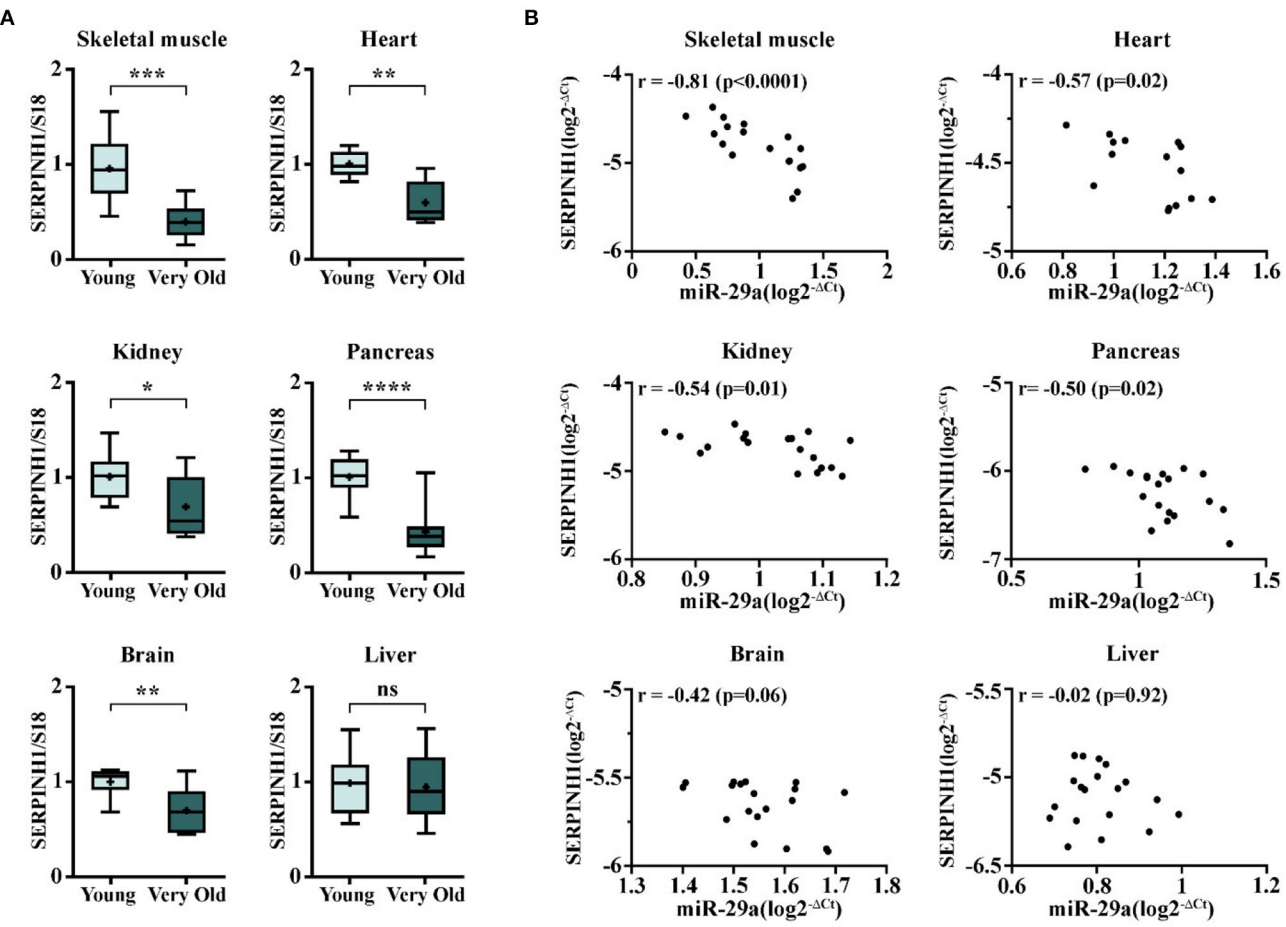
The expression of SERPINH1 and correlation between SERPINH1 and miR-29a expression levels in various organs. **(A)** The expression of SERPINH1 in the heart, the skeletal muscle, the brain, the kidney, the pancreas, and the liver. For each organ, the expression is presented relative to the young group. *N* = 5–10 animals/group; *p < 0.05, **p < 0.01, ***p < 0.005, and ****p < 0.001. **(B)** Pearson's correlations between SERPINH1 and miR-29a expression levels in the six organs mentioned above.

To further validate the biological link between miR-29a and SERPINH1 synthesis *in vivo*, the transcriptional levels of SERPINH1 were measured in various organs from very old mice (24–25-months-old) relative to their young counterparts. Quantitative RT-PCR analysis revealed that SERPINH1 was decreased in all aged organs, except the liver (Figure 5A). Noteworthy, the expression level of miR-29a inversely correlated with SERPINH1. The strongest correlation was found in the skeletal muscle, whereas the heart, the kidney, and the pancreas only showed moderate associations. Still, no significant association was found in the brain (Figure 5B). Our results are also concurrent with the data from the Tabula Muris Senis registry, which showed progressive decreases of SERPINH1 mRNA levels in the heart, the limb muscle, the kidney, and the pancreas of the mouse, during aging (https://twc-stanford.shinyapps.io/maca/).

### Other Effects of miR-29a Increase on Muscle Cells

**Figure 6:**
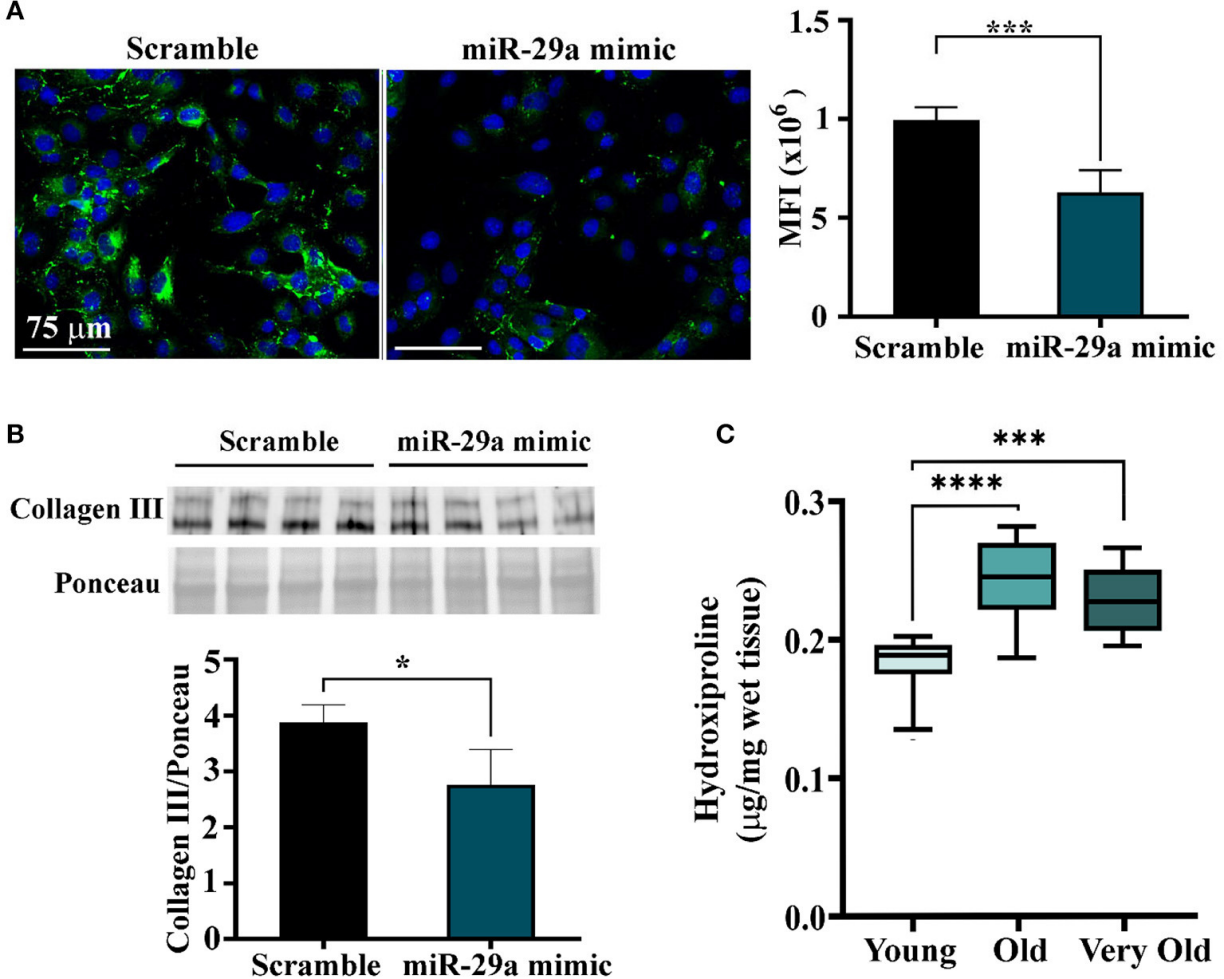
Quantification of collagen proteins in HL-1 cells overexpressing miR-29a and in ventricular lysates obtained from young (2–3-months-old), old (16–18-months-old), and very old (24–25-months-old) animals. **(A)** Immunofluorescence images of HL-1 cells at 24 h after transfection with mir-29a mimic or scramble miRNA. The histogram shows the quantification of the fluorescence signal produced by collagen III staining, expressed as mean fluorescence intensity. ***p < 0.005. **(B)** Western blot analysis of the collagen III in miR-29a-overexpressing HL-1 cells. The histogram shows the densitometry quantification normalized to total protein. *p < 0.05. **(C)** The hydroxyproline levels in tissue lysates of cardiac ventricles were obtained from young, old, and very old animals. ***p < 0.005 and ****p < 0.001.

To further explore the function of miR-29a increase in the old heart and the skeletal muscle, the protein levels of collagen I and III, two targets of this miRNA which are both under the direct control of SERPINH1, were determined in miR-29a-overexpressing HL-1 cells. Immunofluorescence staining followed by fluorescence quantification showed decreased levels of collagen III (Figure 6A) and collagen I (Supplementary Figure 6) in the HL-1cells 1 day after transfection. This data was also validated by Western blot analysis (Figure 6B), which showed a reduced level of collagen III in cell lysates obtained from miR-29a-overexpressing cells when compared to miRNA scramble-transfected cells. However, the quantification of hydroxyproline in the cardiac ventricle lysates obtained from the young, old, and very old animals, as a measure to estimate total collagen levels, showed increased levels of collagens in the old animals (Figure 6C), a result that was not intriguingly, being in agreement with the fibrosis development in the aging heart. This data suggests that the increase in the miR-29a level, noticed in multiple organs in the old individuals, is an adaptive response of the organism to the increased fibrosis associated with the aging process.

**Figure 7:**
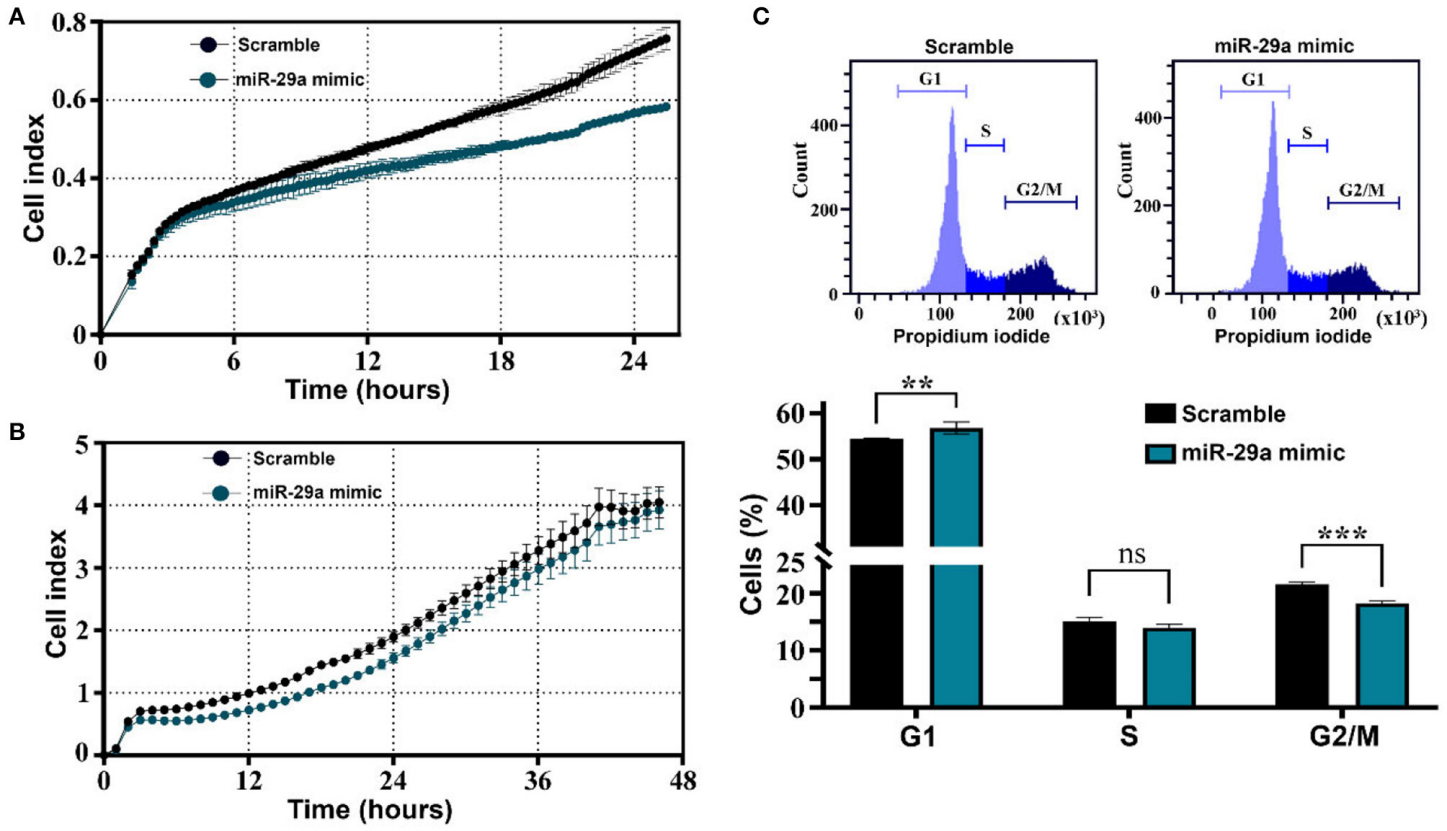
Impact of miR-29a on muscle cell migration and proliferation. **(A)** Real-time profiling of HL-1 cell migration in CIM plates, after transfection with miR-29a mimic or scrambled miRNA control. The data represent one representative experiment of three experiments performed with similar results. **(B)** Real-time profiling of HL-1 cell adherence and proliferation in E-plates, after transfection with miR-29a mimic or scrambled miRNA control. The data represent one representative experiment of three experiments performed in quadruplicates with similar results. **(C)** Cell-cycle distribution and percentage of cells in G0/G1, S, and G2 /M phases. The data represent the mean +/– SD of 3 independent experiments performed. **p < 0.01 and ***p < 0.005.

The effects of miR-29a have been further evaluated in HL-1 cells by examining its effects on cell migration and proliferation. Cell migration was analyzed with the xCELLigence system, by following the capacity of cells to infiltrate the membrane pores of CIM plates in response to the chemoattractant molecules within the serum. The results showed a decreased migration ability of miR-29a overexpressing cells in comparison with the designed control cells (Figure 7A). Moreover, cell proliferation was also affected by miR-29a, as demonstrated by xCELLigence analysis of HL-1 myocytes after transfection (Figure 7B), as well as by cell cycle analysis of cells evaluated at 24 h after transfection initiation (Figure 7C). The results concurred toward a slower proliferation rate of cells overexpressing miR-29a, as illustrated by the decreased cell index and diminished percentage of cells in the G2/M phase, in comparison to scramble-transfected cells. The same effects have been observed on 3T3 fibroblasts cells (data not shown). Considering the activation and proliferation of fibroblasts as common processes accompanying fibrosis development, this data might indicate a protective role of miR-29a upregulation during the natural aging process. However, additional experiments are necessary to confirm the protective role of miR-29a in the aging process, as well as the downregulation of SERPINH1 as a direct target of miR-29a in the old organs.

In conclusion, our data indicate that miR-29a is a potent posttranscriptional repressor of SERPINH1 in multiple organs and its upregulation is a common feature of the aging process, suggesting that it might represent a potential therapeutic target for age-related adverse matrix remodeling and tissue fibrosis.

## Discussion

Deregulation of the miRNA profile in aging has been reported in several studies (40–42), yet only a small number of miRNAs have been directly associated with aging or aging-related cardiac diseases. Fibrosis and hypertrophy are important pathologies in the aged heart and are the primary causes of heart failure (43). Among the miRNAs with documented roles in cardiac diseases, miR-22 was mainly associated with hypertrophy, while miR-18a, miR-21, and miR-29a were associated with fibrosis. The aim of this paper was to evaluate the expression levels of miR-18a, miR-21a, miR-22, and miR-29a in the aging process. Our studies demonstrated that (i) miR-21a, miR-22, and miR-29a are upregulated in the aged heart; (ii) miR-21a and miR-29a are also upregulated in other organs during aging; (iii) miR-29a is highly expressed in many organs and it negatively regulates with the SERPINH1 gene expression level; (iv) a direct negative correlation exists between miR-29a and SERPINH1 expression levels during aging in the cardiac ventricle, the skeletal muscle, the kidney, and the pancreas.

It might be worth mentioning that, although the miR-18a level was not found significantly altered in the heart of old mice (16–18-months-old), its expression slightly, yet significantly, increased in the very old animals (data not shown), indicating that this miRNA gradually changed with aging, but was detected only at very old ages. Similar findings were reported by Schroen's group, which showed an increased expression of miR-18a in the heart of 104-week-old C57Bl/6 mice (22). This increased expression was further associated with the resistance to heart failure, by reducing thrombospondin-1 (THSP-1) and connective tissue growth factor (CTGF), the two direct targets of members of the miR-17–92 cluster (22).

Besides an increase in the size of the old ventricle, the miR-21 and miR-29a were also found in higher levels in other aged organs, including the skeletal muscle and the brain, which are in accordance with other previous studies (44–46). The increase of miR-29 in the skeletal muscle in old rodents resulted in the inhibition of proliferation of muscle progenitor cells, by suppressing the mediators of cell proliferation, IGF-1, p85α, and B-Myb, which in turn led to cellular senescence and contributed to the aging-induced loss of muscle mass and senescence (45). As both miRNAs were found upregulated in the mediastinal lymph nodes, the spleen, and the thymus, we presumed that these changes might be connected to the immune cells infiltrating the organs in advanced age (inflammaging). In addition, this hypothesis was reinforced by the unchanged expression levels of these two miRNAs in cardiac fibroblasts and cardiomyocytes isolated from young and old mice, which made likely the existence of external miRNA sources. According to a recently published comprehensive analysis of aging dynamics, based on RNA-sequencing of 17 organs and plasma proteomics at 10 ages across the mouse lifespan, a widespread immune cell activation became detectable in multiple organs after middle age (47). Therefore, inflammaging may act as both the driver of the whole-organ inflammatory signal, as well as a contributor to the shifts in miRNA levels in aged organs. Interestingly, upregulation of miR-29a in the aged brain was associated with inflammation, by decreasing the expression of two important regulators of microglial activation, such as the IGF-1 and CX3CL1 (44).

Multiple studies documented the increased deposition of ECM proteins in the heart, the pancreas, the kidney, and other organs with advancing age (2, 48, 49), making fibrosis a hallmark of aging. Although both miR-21 and miR-29a have been involved in fibrosis, they appear to have opposite roles. MiR-21 has been associated with the development of fibrosis in patients with pulmonary, hepatic, renal, or cardiac pathologies. Thus, in diabetic cardiomyopathy, an increased level of miR-21 was associated with cardiac interstitial and perivascular fibrosis through NFκB/SMAD7 signaling pathway (8). Other findings showed that miR-21 promoted cardiac fibrosis *via* the TGF-β/Smad7 signaling pathway after myocardial infarction (50) and *via* the CADM1/STAT3 pathway in patients with atrial fibrillation (51). In a transgenic mouse model of cardiac failure, the elevated expression level of miR-21 induced fibrosis, hypertrophy, and cardiac dysfunction, by inhibiting SPRY1, which promoted ERK–MAPK signaling and cardiac fibroblast survival (18). MiR-21 was also found upregulated in the lungs of patients with idiopathic pulmonary fibrosis (52), as well as in kidney fibrosis that followed acute kidney injury or chronic allograft dysfunction (53). Moreover, miR-21 upregulation was correlated with hepatic fibrosis in human cirrhotic liver samples (54).

In contrast, the members of the miR-29 family are known for their anti-fibrotic effects in various organs, by targeting genes involved in ECM production and fibrosis (55). Thus, miR-29 was found downregulated in the border zone of the infarcted myocardium and its expression was correlated with the upregulation of multiple ECM proteins, such as collagen I, II, III, and fibronectin 1 (33). In the mouse model of angiotensin II (Ang II)-induced hypertension, the cardiac fibrosis was abrogated by miR-29b overexpression, which was negatively regulated by TGF-β/Smad signaling in fibrosis (56), while in a model of isoproterenol-induced fibrosis, miR-29a attenuated cardiac fibrosis by targeting DNMT3A expression leading to inhibition of the Ras/ERK1/2 signaling pathway (57). The same mechanisms involving TGF-β/Smad3 signaling were described for miR-29b inhibition in bleomycin-induced pulmonary fibrosis (58) and the development of progressive renal fibrosis in obstructive nephropathy (59). Moreover, the miR-29 family was found downregulated in carbon tetrachloride-induced liver fibrosis in mice, as well as in the livers of patients with advanced liver fibrosis (60).

Although there is substantial evidence for the fact that the miR-29 family abrogates the development of fibrosis by inhibiting collagen synthesis in cardiac fibroblasts, its role in cardiac hypertrophy is still controversial. In a mouse model of cardiac pressure overload, miR-29 was shown to promote pathologic hypertrophy of cardiac myocytes and overall cardiac dysfunction (61). As the expression level of miR-29 in cardiomyocytes isolated from hearts with transverse aortic constriction surpassed those in cardiac fibroblasts, its relevance in the progression of cardiac hypertrophy appeared as more meaningful, thus explaining the overall protecting effect of miR-29 inhibition (or its genetic deletion) on the cardiac hypertrophy and fibrosis in this pathology. The authors also reported that the overexpression of miR-29 in primary cardiomyocytes led to the activation of Wnt signaling, which promoted hypertrophy and the sequential release of profibrotic factors (61). Such discrepancies may be explained by the diversity of mechanisms regulating the miR-29 family, with different stimuli activating distinct signaling pathways and leading to various responses.

A comparative analysis of miR-21a and miR-29a expression levels in various organs tilt the balance in favor of miR-29a, whose expression was much stronger in all analyzed organs. This data suggests that the anti-fibrotic effect of miR-29a prevails in old organs, thus conferring an overall protective anti-fibrotic mechanism in aging.

Besides its anti-fibrotic role, miR-29 was also reported to have protective effects in cancer, elicited by various mechanisms (30). Thus, miR-29 was shown to directly target CDK6 (62) and CDC42 (63), proteins with important roles in controlling cell proliferation in several cancer types. In addition, it was reported that the mantle cell lymphoma patients with downregulated miR-29 showed shorter survival rates compared to patients with relatively higher levels of miR-29 (62). MiR-29a was also shown to inhibit autophagy in pancreatic cancer cells, a mechanism by which the cancer cells survive and acquire chemoresistance. MiR-29a was significantly downregulated in pancreatic cancer cells, yet its overexpression led to increased sensitization and inhibition of autophagy in chemo-resistant cell lines (64). Moreover, miR-29b was found downregulated in ovarian cancer and its restoration led to the inhibition of glycolysis and glucose metabolism in cancer cells by targeting AKT2 and AKT3 (65).

Although miR-29 was found to increase in multiple aged organs in our study, its expression was not changed in the liver. Similar results were found in another report, which analyzed the expression of 367 miRNAs in the aged liver, yet no variations in miR-29 were reported (66). However, it would be worth mentioning that miR-29 was found upregulated in the liver of young mice with Hutchinson-Gilford progeria syndrome, but not in the naturally aged mice (67).

Pathway enrichment analysis of target genes of miR-29a revealed SERPINH1 as a major player in connective tissue homeostasis. SERPINH1, also named Hsp47 (Heat-shock protein 47), is a molecular chaperone that belongs to the Serpin family, yet has no serin protease inhibitory effect. SERPINH1 is found primarily in the secretory pathway of collagen-producing cells, where it binds to and stabilizes procollagen, preventing incomplete folding and lateral aggregation prior secretion (68–70). Without the assistance of SERPINH1, the type I collagen is unable to form the rigid, protease-resistant, and triple-helical structure (71). The importance of SERPINH1 in collagen secretion, processing, and fibril assembling, as well as its deposition in the ECM, had been demonstrated in multiple studies (72, 73). In the human breast cancer tissue, SERPINH1 was positively correlated with collagen I, collagen IV, and fibronectin expression (72). Mutation of SERPINH1 was associated with a severe phenotype of Osteogenesis Imperfecta, in which the secreted type I collagen structure was compromised and became sensitive to protease activity (73). Moreover, knockout mice presented abnormal epithelial tissues, ruptured blood vessels, discontinued basement membranes; they did not survive beyond 11.5 days post-coitus (71).

In this paper, we report that the overexpression of miR-29a in HL-1 cells led to SERPINH1 downregulation, thus endorsing SERPINH1 as a direct target of miR-29a. Furthermore, we showed that miR-29a negatively correlated with SERPINH1 expression levels in various organs during aging. To the best of our knowledge, this is the first report showing that SERPINH1 is downregulated in aged organs, likely as a consequence of the increase in miR-29a level. The presence of miR-29a-binding site within SERPINH1- 3' UTR was previously validated *via* luciferase reporter assay and western blot (74). Besides, the downregulation of miR-29 was found to be associated with the upregulation of SERPINH1 in various cancers (glioma, cervical, breast, and renal), being involved in the growth, proliferation, migration, and invasion of cancer cells. Consequently, the suppression of SERPINH1 by miR-29- overexpression canceled the progression of cancers (72, 74–77). In good agreement with these studies, we reported here that the overexpression of miR-29a inhibited the proliferation and migration of HL-1 cardiac muscle cells and 3T3 fibroblasts cells. Considering that fibroblast activation and proliferation are important processes leading to cardiac fibrosis (78), this data might indicate a protective role of miR-29a upregulation during the natural aging process. However, additional experiments are necessary to confirm the protective role of the miR-29a/SERPINH1 axis in the aging process.

Recent studies showed that SERPINH1 increased in mouse cardiac endothelial cells and this increase was associated with endothelial dysfunction and impaired vascular remodeling (79). Moreover, multiple studies showed that SERPINH1 was overexpressed in fibrosis-related diseases. In Fischer 344 rats, which spontaneously develop kidney fibrosis in aging, the overexpression of SERPINH1 was associated with increased synthesis of collagens I, III, and IV (80). The same results were observed in carbon tetrachloride-induced liver fibrosis and bleomycin-induced pulmonary fibrosis (81, 82). Additionally, cardiac fibrosis after pressure overload injury was also associated with the increased levels of SERPINH1, collagen I, III, and V and, moreover, myofibroblast-specific deletion of SERPINH1 reduced fibrosis and cardiac hypertrophy (83). The SERPINH1 was also associated with cardiac fibrosis following myocardial infarction. Thus, rats with ligated left anterior descendant coronary artery showed upregulated levels of SERPINH1 in the infarcted zone, which were maintained for up to 28 days after the intervention (84). Moreover, administration of SERPINH1 antisense oligonucleotides reduced cardiac remodeling and improved cardiac function in a rat model of myocardial infarction (85). These findings strengthen our hypothesis that upregulation of miR-29a during natural aging represents a protective mechanism against fibrosis by targeting SERPINH1.

Although collagen level is elevated in fibrosis-related pathologies, multiple studies showed that its increased synthesis was not the primary cause of fibrosis in aging. Contrarily, several studies reported that mRNA expression of collagens I and III was even reduced in aged rat myocardium (86, 87). Important sources of collagen accumulation in aging also result in the attenuation of matrix-degrading pathways (88), as well as the accumulation of advanced glycation end products, which caused collagen cross-linking (89). In this paper, we found increased amounts of hydroxyproline in the cardiac ventricles harvested from old and very old mice, in comparison to their young counterparts. Our results are in accordance with other reports showing that cardiac fibrosis was associated with the aging process. A possible explanation for increased collagen levels is the upregulation of miR-29a and subsequent downregulation of SERPINH1. Thus, miR-29a concomitantly regulates the transcriptional levels of collagen proteins, as well as collagen maturation process (by targeting the specific collagen chaperon, SERPINH1). Our results suggest that miR-29a increase in multiple organs in old individuals develops as an adaptive response of the organism to the increased fibrosis associated with the natural aging process. However, further studies are necessary to confirm the protective role of miR-29a in the aging process.

In conclusion, our study demonstrates that miR-29a is highly expressed in many organs and its expression is further upregulated during the natural aging process. This process occurs in association with the downregulation of SERPINH1, which thus might be a potential therapeutic target for adverse matrix remodeling and aging-associated tissue fibrosis.

## Data Availability Statement

The raw data supporting the conclusions of this article will be made available by the authors, without undue reservation.

## Ethics Statement

The animal study was reviewed and approved by National Sanitary Veterinary and Food Safety Authority (nr 389/22.03.2018).

## Author Contributions

ER-N, A-ML, MP, CM, and CN performed experiments. ER-N and AB designed the work, analyzed and interpreted the data, and wrote the manuscript. AB gave the final approval of the manuscript. All authors contributed to the article and approved the submitted version.

## Funding

This work was supported by the Romanian Ministry of Education, PN-III-P4-ID-PCE-2020-1340-contract 122/2021.

## Conflict of Interest

The authors declare that the research was conducted in the absence of any commercial or financial relationships that could be construed as a potential conflict of interest.

## Publisher's Note

All claims expressed in this article are solely those of the authors and do not necessarily represent those of their affiliated organizations, or those of the publisher, the editors and the reviewers. Any product that may be evaluated in this article, or claim that may be made by its manufacturer, is not guaranteed or endorsed by the publisher.
