# Supplementary material for: MiR-29a Increase in Aging May Function as a Compensatory Mechanism Against Cardiac Fibrosis Through SERPINH1 Downregulation
Source: Front Cardiovasc Med. 2022 Jan 18;8:810241. doi: 10.3389/fcvm.2021.810241 (PMC8804242; doi:10.3389/fcvm.2021.810241)
Supplement: Supplementary file 1 [file Data_Sheet_1.PDF]

Supplemental materials

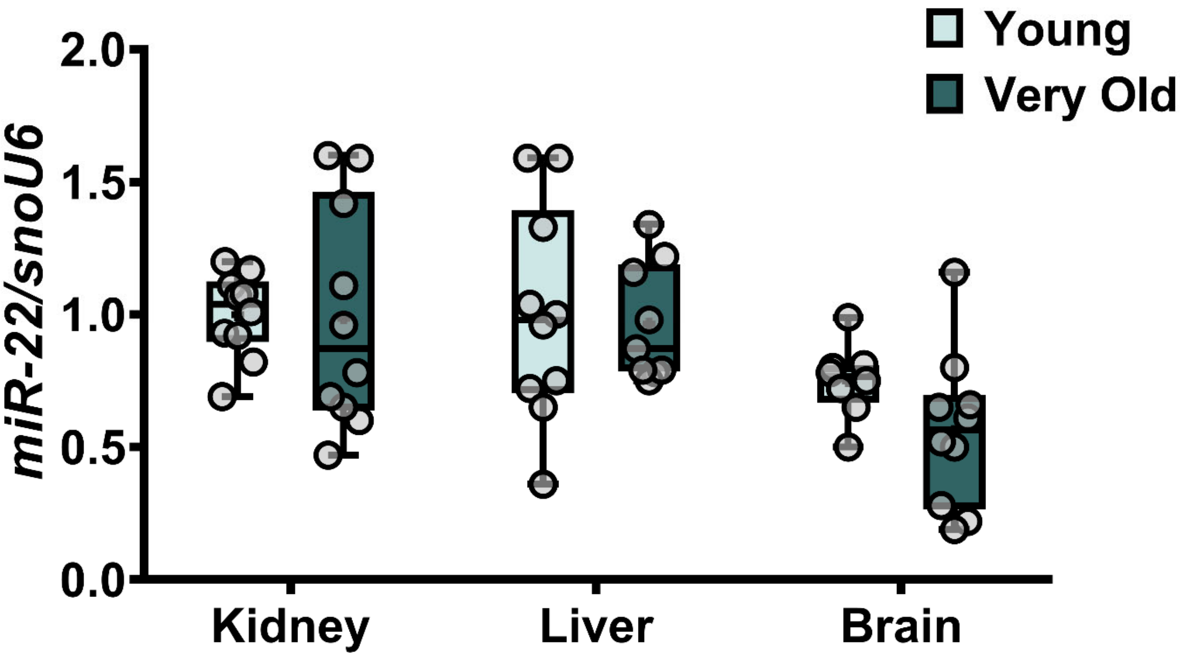

Supplemental figure 1. Aged-related variations in the expression level of miR-22 in the kidney, liver and brain in C57Bl/6 mice. n=10 animals/group.

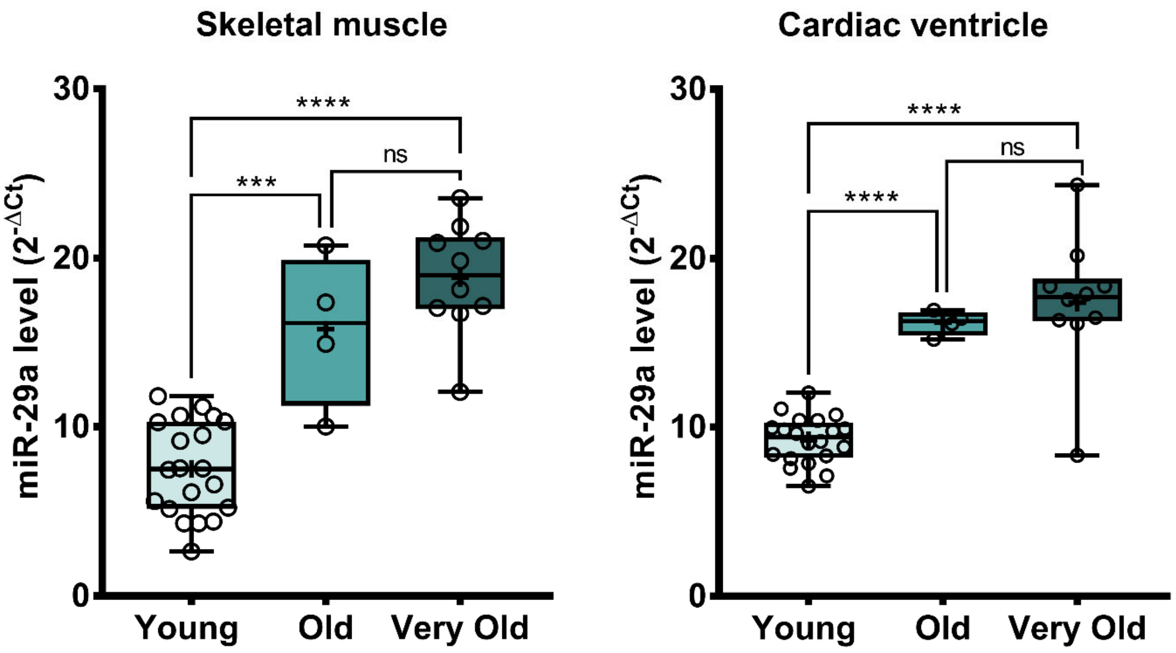

Supplemental figure 2. MiR-29a expression levels in skeletal muscle and cardiac ventricle at young (2-3-month-old, n=20), old (16-18-month-old, n=4) and very old (24-25-month-old, n=10) mice. The histograms were obtained using the data from two experimental lots analyzed at one-year interval.

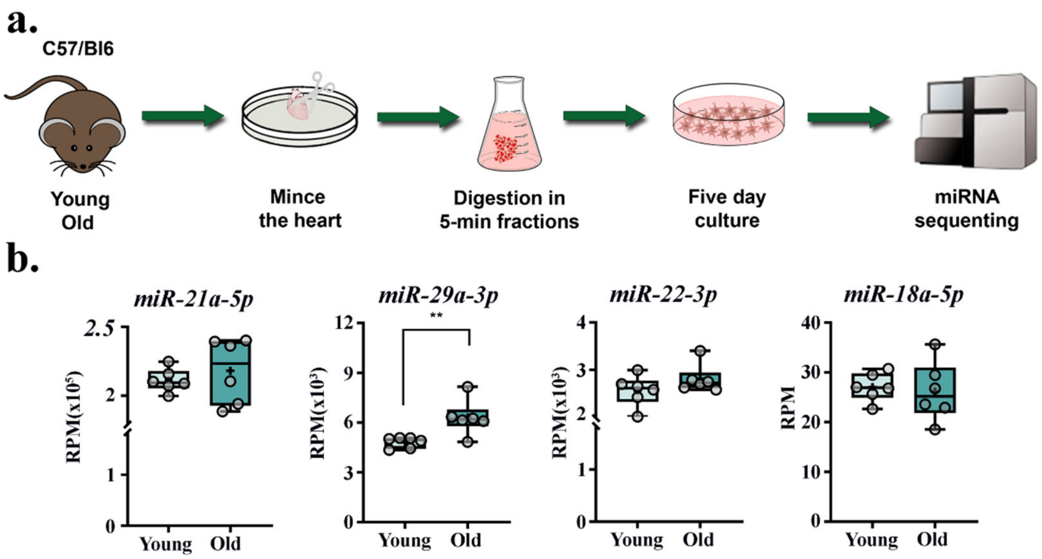

Supplemental figure 3. Aged-related changes in the expression level of miR-18a, miR-21a, miR-22 and miR-29a in the cardiac fibroblasts. (a) Schematic illustration of the experimental design. (b) The expression of miRNAs in old and young cardiac fibroblasts, generated by next generation sequencing analysis. RPM, reads per million. The differential expression analysis with edgeR v3.28 used the quasi-likelihood negative binomial generalized log-linear model functions provided by the package, as previously detailed (Lupan et al., 2021).

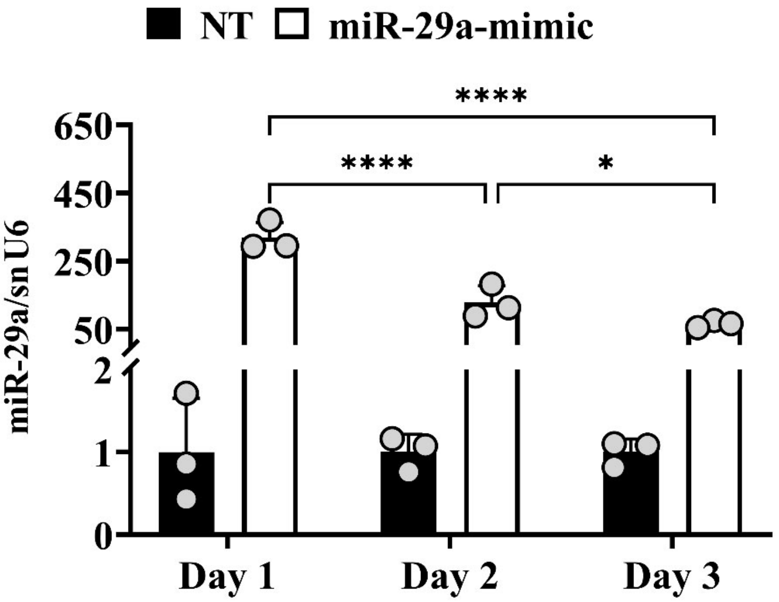

Supplemental figure 4. The time-dependent variations in the expression level of miR-29a in HL-1 cells, after transfection with miR-29a mimic or scramble miRNA. snRNA U6 was used for data normalization (n=3).

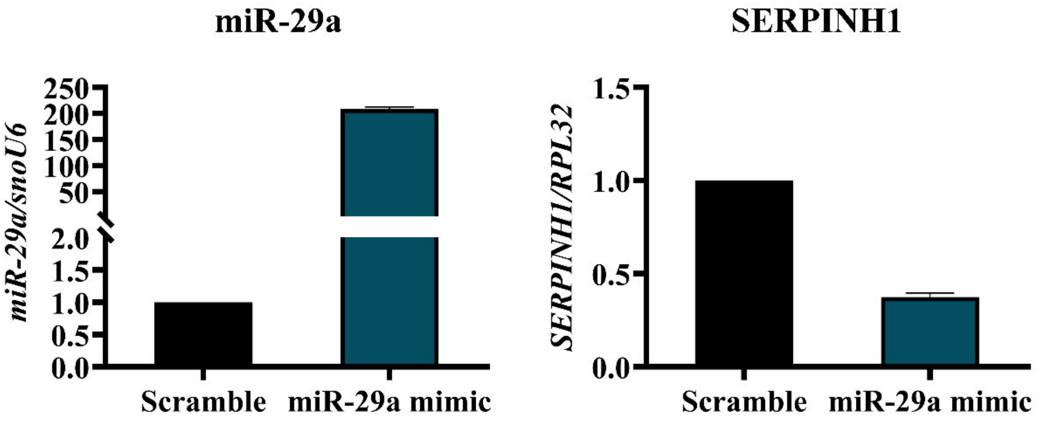

Supplemental figure 5. (left) The expression level of miR-29a in 3T3 cells, evaluated at 24 hours after transfection with miR-29a mimic or scramble miRNA. snRNA U6 was used for data normalization. (right) The expression of SERPINH1 mRNA level in 3T3 cells at 24 hours after transfection of cells with miR-29a mimic.

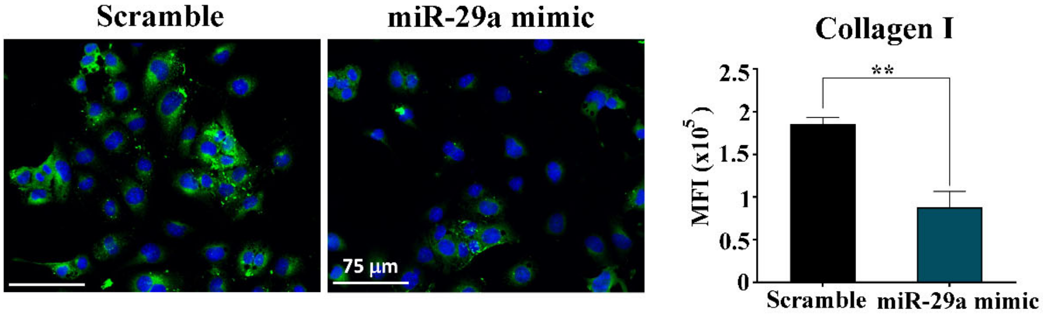

Supplemental figure 6. (left) Immunofluorescence images of collagen I protein in HL-1 cells at 24 hours after transfection with mir-29a mimic or scramble miRNA. The histogram shows the quantification of the fluorescence signal, expressed as mean fluorescence intensity. ** $p < 0.01$.
